# Social vulnerability of children and adolescents who are victims of sexual violence assisted in a specialized service: descriptive study, Porto Alegre and metropolitan region, 2018

**DOI:** 10.1590/S2237-96222026v35e20240780.en

**Published:** 2026-10-30

**Authors:** Beatriz Raffi Lerm, Vanessa Pletsch, Maria De Fátima Fernandes Géa, Iná da Silva dos Santos, Andrea Pereira Regner, Camila Giugliani

**Affiliations:** 1 Universidade Federal do Rio Grande do Sul, Porto Alegre, RS, Brazil; 2 Hospital Materno Infantil Presidente Vargas, Porto Alegre, RS, Brazil; 3 Universidade Federal de Pelotas, Pelotas, RS, Brazil

**Keywords:** Child Abuse, Sexual, Sex Offenses, Geographic Mapping, Adolescent, Cross-Sectional Studies., Abuso Sexual Infantil, Delitos Sexuales, Mapeo Geográfico, Adolescente, Estudios Transversales

## Abstract

**Objective:**

To describe the profile and social context of children and adolescents who are victims of sexual violence, as well as their territories of residence, assisted in 2018 by a referral service in Porto Alegre and its metropolitan region.

**Methods:**

Descriptive study with individual- and aggregate-level analyses using geoprocessing. The Social Vulnerability Index (SVI) and the Human Development Index (HDI) of the Human Development Units (UDH) of the victims' residences were assessed. At the individual level, the indices were compared with the mean of the UDH of Porto Alegre and its metropolitan region. At the aggregate level, the rate of cases assisted per 1,000 inhabitants aged <18 years was calculated, and Spearman's correlation and a choropleth map were used to visualize the results.

**Results:**

A total of 1,519 children and adolescents were included, predominantly girls (77.7%) and victims of intrafamilial violence (53.9%). Regarding the UDH mean, 63.0% lived in areas with higher SVI, and 68.5% lived in areas with lower HDI. At the aggregate level, the case rate did not correlate with the indices analyzed. A weak correlation was observed between the HDI and the number of cases (r=0.095; p-value<0.001).

**Conclusion:**

At the individual level, most victims resided in more vulnerable locations with lower human development. At the aggregate level, the results did not indicate a pattern. The findings aim to contribute to addressing sexual violence, taking into account territories and the social determination of health.

## Resumen

**Objetivo:**

Describir el perfil y el contexto social de niños, niñas y adolescentes víctimas de violencia sexual, así como sus territorios de residencia, atendidos en 2018 por un servicio de referencia en Porto Alegre y su región metropolitana.

**Métodos:**

Estudio descriptivo con análisis a nivel individual y agregado mediante el geoprocesamiento. Se evaluaron el Índice de Vulnerabilidad Social (IVS) y el Índice de Desarrollo Humano (IDH) de las Unidades de Desarrollo Humano (UDH) de residencia de las víctimas. A nivel individual, los índices se compararon con el promedio de las unidades de desarrollo humano de Porto Alegre y de su región metropolitana. A nivel agregado, se calculó la tasa de casos atendidos por 1.000 habitantes <18 años y se utilizó la correlación de Spearman y la representación mediante mapa coroplético.

**Resultados:**

Se incluyeron 1.519 niños, niñas y adolescentes, predominantemente niñas (77,7%) y víctimas de violencia intrafamiliar (53,9%). En relación con el promedio de las unidades de desarrollo humano, el 63,0% residía en áreas con mayor índice de vulnerabilidad social y el 68,5% en áreas con menor índice de desarrollo humano. A nivel agregado, la tasa de casos no se correlacionó con los índices analizados. Se observó una correlación débil entre el índice de desarrollo humano y el número de casos (r=0,095; valor p<0,001).

**Conclusión:**

A nivel individual, la mayoría de las víctimas residía en lugares más vulnerables y con menor desarrollo humano. A nivel agregado, los resultados no indicaron un patrón. Los hallazgos buscan contribuir al enfrentamiento de la violencia sexual, considerando los territorios y la determinación social de la salud.

**Ethical aspects**

This research respected ethical principles, having obtained the following approval data: Research ethics committee: Hospital Materno Infantil Presidente Vargas. Opinion number: 27707119.1.0000.5329. Approval date: 12/2/2020. Certificate of submission for ethical appraisal: 3,835,179. Informed consent record: Exempt.

## Introduction 

Sexual violence, in addition to constituting a violation of human rights, causes psychological, emotional, and physical sequelae in victims, both in the short and long term ^1^, especially when it occurs during the early stages of life ^2^. In the child and adolescent context, the term sexual violence refers to any act or sexual play intended to sexually stimulate the child or adolescent, to obtain sexual gratification, carried out by perpetrators who are at a more advanced stage of psychosexual development than the child or adolescent ^3^.

According to the ecological model proposed by the World Health Organization (WHO), the causes of sexual violence involve various factors, including community-level factors, such as poverty and community tolerance of sexual violence events; and social-level factors, such as machismo, gender inequity, and social norms that normalize sexual violence ^1^ ^,^ ^4^. 

Sexual violence against minors most often occurs within the nuclear family, which contributes to resistance in reporting the events ^3^. Research on this topic is particularly complex due to ethical considerations, methodological challenges, and associated taboos ^1^, as well as underreporting of cases ^5^. 

According to criminal records from 2023, Rio Grande do Sul ranked tenth out of the 26 Brazilian states for rape of individuals under 18 years, with an estimate of 174 cases per 100 thousand inhabitants ^6^. Since 2011, the reporting of violence by health services has been mandatory through the Violence and Accident Surveillance System (*Sistema de Vigilância de Violências e Acidentes*, VIVA); however, noncompliance among professionals remains frequent ^7^. An estimated 4.2% of rape cases in Brazil are recorded in this system ^8^. 

To approximate the reality of the magnitude of child and adolescent sexual violence, it is necessary to use different data sources, such as police reports ^9^, specialized services ^10^, and official databases ^7^.

Although still representing a serious public health problem in Brazil, progress has been made over the last decade, such as Law No. 13,431/2017, which established the System for the Guarantee of Rights of Children and Adolescents (*Sistema de Garantia de Direitos da Criança e do Adolescente*, SGDCA) who are victims or witnesses of violence, integrating health, social assistance, public safety, and justice ^11^. Within the scope of this legislation, specialized services are also foreseen to prevent revictimization.

Most Brazilian health studies on sexual violence against children and adolescents are based on Notifiable Diseases Information System (*Sistema de Informação de Agravos de Notificação*, SINAN) data and focus on characterizing the profile of victims, perpetrators, and circumstances of the violence ^8^. Studies investigating the social contexts of children and adolescents who are victims of this violence are still scarce. Geoprocessing can help explore the relationship between sexual violence and social vulnerability ^12^, which may contribute to the public health decision-making process ^13^.

This study aims to describe the profile and social context of children and adolescents who are victims of sexual violence, as well as their territories of residence, assisted in 2018 by a referral service in Porto Alegre and its metropolitan region.

## Methods

Study design

Descriptive study using health service registry data, with individual- and aggregate-level analyses, employing geoprocessing of data. Data were presented in accordance with the guidelines for observational studies ^14^.

Setting

Data from medical records of a service dedicated to the biopsychosocial care of children and adolescents who were victims of sexual violence were used. This service, headquartered in Porto Alegre, Rio Grande do Sul, was one of the pioneers in providing comprehensive care to victims-it conducts police reports, forensic examinations, biopsychosocial care, and other referrals to ensure continuity of care within the protection network-to prevent revictimization through repeated interviews at different institutions ^15^.

Participants

Medical record data for children and adolescents assisted by the service were extracted, focusing only on care provided in 2018. Cases in which the victim did not reside in Porto Alegre or in the metropolitan region of the capital were excluded.

Data collection and data sources

The variables sex, date of birth, place of residence, and relationship with the abuser were collected in 2020 from electronic medical records. Data were entered twice into a Microsoft Excel 2016 spreadsheet (version 16.0) and coded to preserve the confidentiality of the individuals involved. 

Variables

Age, expressed as a fractional number of years, was calculated as the difference between the date of care and the date of birth. Based on this measure, five age group categories in years were generated (<1; 1-5; 6-9; 10-14; 15-18). The sex variable was categorized as female and male. Regarding place of residence, a dichotomous variable was generated distinguishing children residing in Porto Alegre from those living in its metropolitan region. The victim's relationship with the perpetrator of violence was categorized as: intrafamilial (father, mother, stepfather, stepmother, sibling, grandfather/grandmother, grandmother's partner, uncle/aunt, brother-in-law/sister-in-law, cousin, great-grandfather/great-grandmother, nephew/niece, and mother's stepchild); extrafamilial (boyfriend/girlfriend, friend, acquaintance, or peer at the social institution where the victim lived-foster care institutions, shelters, foster homes, and orphanages); intra- and extrafamilial (when the same child had at least one perpetrator from each of the aforementioned groups); and undefined (when no perpetrator was indicated). Two dichotomous variables were generated: one referring to the number of perpetrators involved, distinguishing victims who reported one perpetrator from those who reported two or more perpetrators; and another indicating whether the violence was intrafamilial. The SVI is calculated as the arithmetic mean of the indices for the dimensions of urban infrastructure, human capital, and income and labor. This index ranges from 0 to 1, and it is important to note that it has negative polarity: the closer to 1, the greater the social vulnerability, that is, the worse the observed conditions. The HDI, in turn, is obtained through the geometric mean of the income, education, and longevity indices and also ranges from 0 to 1. Unlike the SVI, the HDI has positive polarity, indicating that values closer to 1 reflect better human development conditions ^16^.

Although the SVI and HDI have cutoff points and categories previously defined by their developers, in this study, they were classified into quintiles based on values for all Human Development Units (*Undiades de Desenvolvimento Humano*, UDH) in Porto Alegre and its metropolitan region. The classification into quintiles was chosen because it better indicates disparities in these locations, given that the indices are calculated at the national level. 

The SVI presented the following cutoff points: 1st quintile (≤0.176), 2nd quintile (>0.176 to ≤0.230), 3rd quintile (>0.230 to ≤0.286), 4th quintile (>0.286 to ≤0.344), and 5th quintile (>0.344). HDI quintiles were defined as: 1st quintile (≤0.644), 2nd quintile (>0.644 to ≤0.704), 3rd quintile (>0.704 to ≤0.739), 4th quintile (>0.739 to ≤0.828), and 5th quintile (>0.828). 

Two dichotomous variables were constructed to indicate whether the victim resided in a UDH with SVI and HDI indices above the mean for all UDH in Porto Alegre and its metropolitan region, with SVI equal to 0.234 and HDI equal to 0.746.

Geoprocessing

This stage of the analysis was performed using the geospatial processing library GeoPandas, in the Python programming language (available at: https://geopandas.org/). Initially, the exact coordinates of the victims' addresses were obtained using the Google Maps geocoding service. Based on these coordinates, the respective UDH areas were identified. These units are geographic delimitations of capitals and their metropolitan areas, developed on the basis of socioeconomic homogeneity and defined according to the 2010 census tracts of the Brazilian Institute of Geography and Statistics (*Instituto Brasileiro de Geografia e Estatística*, IBGE).

The geospatial delimitation of these areas and municipalities, as well as the respective estimates of SVI, HDI, and the population aged <18 years, are available on the digital platform http://ivs.ipea.gov.br by the Institute for Applied Economic Research (*Instituto de Pesquisa Econômica Aplicada*, Ipea). The UDH was used because it is the most disaggregated level available from Ipea, allowing a closer approximation to socioeconomic reality and capturing relevant intra-urban inequalities. 

Subsequently, a choropleth map at the UDH level was generated, with a color gradient indicating the magnitude of the SVI. The rate of cases assisted per 1 thousand inhabitants aged <18 years in each unit was plotted, and the point size varied with the rate. UDH that had no cases assisted were represented on the map (Figure 1) with hatched shading. A map with HDI values was produced and made available in the supplementary material (Supplementary Figure 1)

Data availability

At the individual level, cases of intrafamilial violence (exclusive or in combination with extrafamilial violence) were described separately. The age of victims was reported as the mean and standard deviation (SD). To compare the mean age between sexes, Student's t-test was used. The association between dichotomous and categorical variables was assessed using Fisher's exact test.

At the UDH and municipal levels, the rate of children and adolescents assisted in 2018 was calculated by dividing the number of cases assisted in each UDH by the local population aged <18 years, then multiplying the result by 1 thousand inhabitants. 

At the UDH level, the variables case rates per 1 thousand inhabitants, number of cases, population aged <18 years, SVI, and HDI were correlated using Spearman's rank correlation, given their asymmetric distributions. The level of significance was set at p-value<0.050. The statistical software used was Stata, version 16.1.

**Figure 1:**
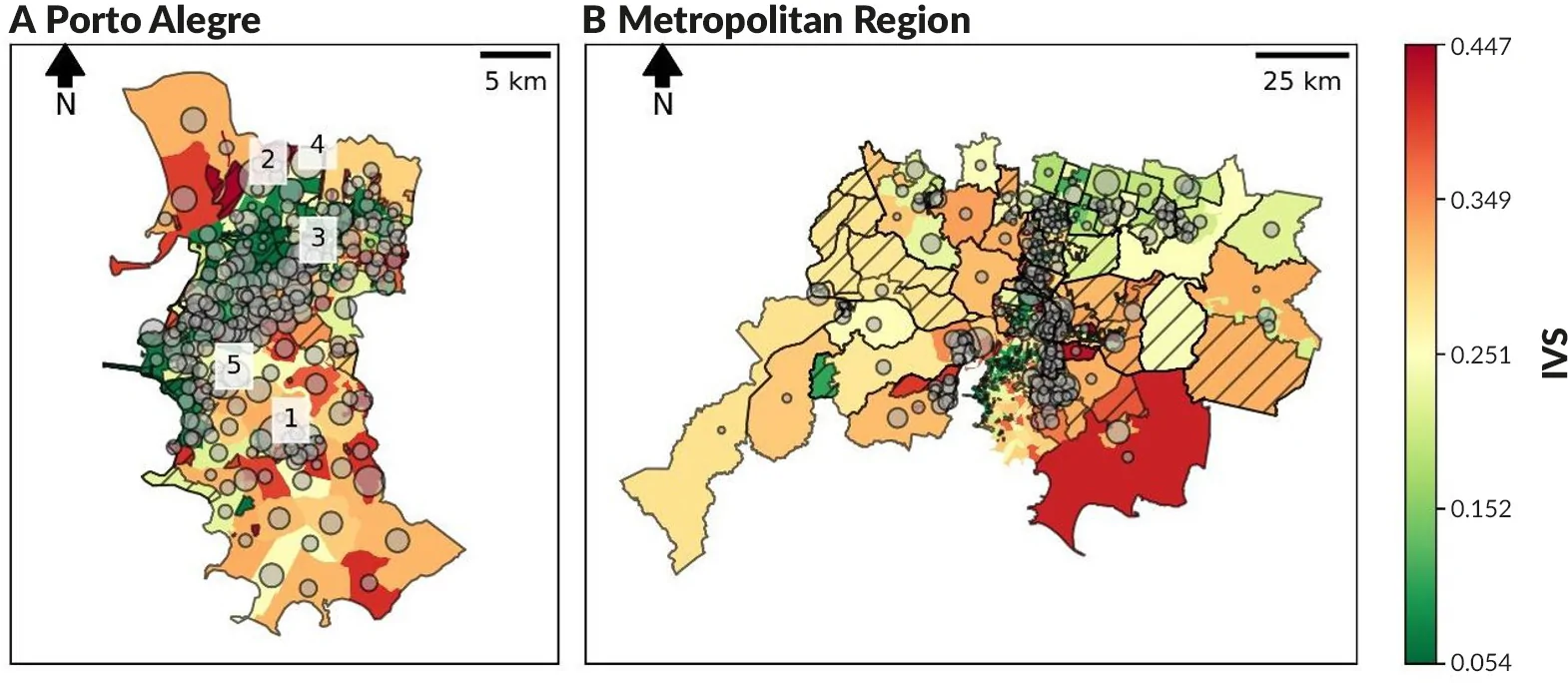
Case rate per 1 thousand inhabitants aged <18 years, by Human Development Unit (UDH) and their respective Social Vulnerability Index (SVI) values in Porto Alegre (A) and Metropolitan Region (B). Porto Alegre and the Metropolitan Region, 2018

Note: Gray circles represent the rate of cases assisted per 1,000 inhabitants aged <18 years in the Human Development Units (UDH), with size varying according to the magnitude of the rates. The color gradient of each UDH corresponds to its SVI value, as indicated in the color legend on the right side of the figure. UDH with no cases assisted is represented with hatched shading. Black outlines indicate municipal boundaries. The numbers on the map indicate the region where the five human development units with the highest case rates are located, all of them belonging to Porto Alegre, listed below: 1- Restinga: Vale do Salso; 2- Navegantes: DC Navegantes/Casa de Passagem; 3- Vila Jardim: Beco Um Salvador Pinheiro/Souza Lobo; 4- Anchieta (district); 5- Vila Nova: Condomínio Cristal II (Porto Alegre).

## Results 

In 2018, a total of 1,913 children and adolescents were assisted at the health service under study. Of these, 1,519 resided in Porto Alegre or its metropolitan region and comprised the final study sample. Among those excluded, 379 were from other municipalities, and ten had no address information in their records. For five individuals, it was not possible to locate the address through geoprocessing analysis. The mean age of the study population was ten years (SD 4.26). The mean age of girls was 10.3 years (SD 4.29), higher than that of boys at 8.8 years (SD 3.93; p-value<0.001). The predominant age group was 10-14 years (40.9%).

In 42 cases (2.8%), two or more perpetrators were involved in the act of sexual violence. Intrafamilial violence was the most frequent type in the study population (53.3%). The proportion of girls who experienced intrafamilial sexual violence was higher (56.3%) compared with boys (43.1%) (p-value<0.001). 

Most victims (63.0%) resided in regions with SVI values above the state estimate, and 31.5% lived in areas with HDI values above those of the remaining UDH. 

A higher proportion of victims was observed in the two highest SVI quintiles (47.8%), with no statistically significant differences between sexes. The highest proportions in relation to HDI were observed in the second and third quintiles (47.3%). A statistically significant difference between sexes was identified (p-value 0.002): the highest proportion of girls was found in the second quintile (25.9%), whereas among boys the quintile with the highest human development predominated (22.4%) (Table 1).

**Table 1:** Frequency (n) and percentage (%) of sociodemographic characteristics and characteristics of the sexual violence event among children and adolescents assisted at the health service. Porto Alegre and the Metropolitan Region (MR), 2018 (n=1,519)

Characteristics	Total	Female (n=1,180)	Male (n=339)	p-value^a^
	n (%)	n (%)	n (%)	
Age group (years)				<0.001
<1	7 (0.5)	5 (0.4)	2 (0.6)	
1-5	342 (22.5)	247 (20.9)	95 (28.0)	
6-9	363 (23.9)	252 (21.4)	111 (32.7)	
10-14	622 (40.9)	511 (43.3)	111 (32.7)	
15-18	185 (12.2)	165 (14.0)	20 (5.9)	
Place of residence				0.020
Porto Alegre	770 (50.7)	581 (49.2)	189 (55.8)	
Metropolitan region	749 (49.3)	599 (50.8)	150 (44.2)	
Number of perpetrators involved in the perpetration of sexual violence				0.347
One	1,477 (97.2)	1,150 (97.5)	327 (96.5)	
Two or more	42 (2.8)	30 (2.5)	12 (3.5)	
Relationship with the perpetrator of sexual violence				<0.001
Intrafamilial	810 (53.3)	664 (56.3)	146 (43.1)	
Extrafamilial	362 (23.8)	262 (22.2)	100 (29.5)	
Intra- and extrafamilial	9 (0.6)	7 (0.6)	2 (0.6)	
Undefined	338 (22.3)	247 (20.9)	91 (26.8)	
Social Vulnerability Index (SVI) quintiles				0.268
1^st^	267 (17.6)	196 (16.6)	71 (20.9)	
2^nd^	277 (18.2)	212 (18.0)	65 (19.2)	
3^rd^	249 (16.4)	192 (16.3)	57 (16.8)	
4^th^	362 (23.8)	291 (24.6)	71 (20.9)	
5^th^	364 (24.0)	289 (24.5)	75 (22.2)	
Human Development Index (HDI) quintiles				0.002
1^st^	289 (19.1)	215 (18.2)	74 (21.8)	
2^nd^	368 (24.2)	306 (25.9)	62 (18.3)	
3^rd^	351 (23.1)	280 (23.7)	71(21.0)	
4^th^	257 (16.9)	201 (17.1)	56 (16.5)	
5^th^	254 (16.7)	178 (15.1)	76 (22.4)	
SVI>mean of Porto Alegre and Metropolitan Region (MR)	957 (63.0)	757 (64.1)	200 (59.0)	0.085
HDI≤mean of Porto Alegre and MR	1,041 (68.5)	833 (70.6)	208 (61.4)	0.001

a Fisher's exact test.

Among victims of intrafamilial violence, 56.5% resided in the metropolitan region (p-value<0.001). Half of the individuals belonged to the two quintiles with the highest SVI values (p-value 0.013); regarding HDI, the highest proportion of individuals belonged to the second quintile (p-value<0.001) (Table 2). 

Of the 33 municipalities in the Porto Alegre metropolitan region, 32 recorded care at the health service. Only one municipality, Glorinha, was not represented in the analyses due to the absence of cases during the study period. Of the total 722 UDH in Porto Alegre and its metropolitan region, 296 (40.9%) did not appear in this analysis. Of these, 128 (43.2%) belonged to Porto Alegre. 

Correlation analysis showed statistical significance (p-value<0.001) between the rate of cases assisted and the number of cases (r=0.371), between the rate of cases assisted and the population aged <18 years (r=-0.565), between the number of cases assisted and the population aged <18 years (r=0.498), and between the number of cases assisted and the SVI (r=0.095), as shown in the correlation matrix (Table 3). No significant correlation coefficient was found between SVI values and the case rate of each UDH (r=0.010; p-value 0.843), nor with HDI values (r=0.072; p-value 0.135). 

According to this finding, the map (Figure 1) shows that the largest circles-representing higher case rates-were not always located in UDH areas with higher SVI. The five UDH with the highest case rates are located in Porto Alegre and are indicated on the map by numbers one to five. 

The Restinga/Vale do Salso unit had the highest rate (20.83 cases per 1 thousand inhabitants) and is characterized as a region of high vulnerability and low human development (Table 4). The UDH with the lowest rate (0.15 cases per 1 thousand inhabitants) was Elisa/Santa Fé/Olinda, in Gravataí, which presented higher SVI and lower HDI than the averages for the other UDH. At the municipal level, the highest rate was observed in Igrejinha (4.05 cases per 1 thousand inhabitants), followed by Porto Alegre (2.52 cases per 1 thousand inhabitants). The lowest rates were recorded in Gravataí (0.13 cases per 1 thousand inhabitants) and Canoas (0.29 cases per 1 thousand inhabitants).

**Table 2:** Frequency (n) and percentage (%) of sex and social context of children and adolescents who were victims of intrafamilial sexual violence and assisted at the health service. Porto Alegre and the Metropolitan Region, 2018 (n=819)

Groups	n (%)	p-value^a^
Sex		<0.001
Female	671 (81.9)	
Male	148 (18.1)	
Place of residence		<0.001
Porto Alegre	356 (43.5)	
Metropolitan region	463 (56.5)	
Social Vulnerability Index (SVI) quantiles		0.013
1st	124 (15.1)	
2nd	162 (19.8)	
3rd	124 (15.1)	
4th	197 (24.1)	
5th	212 (25.9)	
Human Development Index (HDI) quintiles		0.001
1st	163 (19.9)	
2nd	214 (26.1)	
3rd	185 (22.6)	
4th	149 (18.2)	
5th	108 (13.2)	

a Fisher's exact test.

**Table 3:** Correlation matrix between the rate of cases assisted per 1,000 inhabitants aged <18 years, number of cases assisted, population aged <18 years, Social Vulnerability Index (SVI), and Human Development Index (HDI) at the Human Development Unit (UDH) level. Porto Alegre and Metropolitan Region, 2018 (n=426)

	Case rate	Number of cases	Population aged <18 years	SVI	HDI
Case rate	1.000				
Number of cases	0.371^a^	1.000			
Population aged <18 years	-0.565^a^	0.498^a^	1.000		
SVI	0.010	0.095^a^	0.044	1.000	
HDI	0.072	-0.056	-0.093	-0.817^a^	1.000

a p-value<0.001.

**Table 4:** Municipalities and Human Development Units (UDH) with the highest and lowest rates of cases assisted per 1 thousand inhabitants aged <18 years, along with their respective Social Vulnerability Index (SVI) and Human Development Index (HDI) values. Porto Alegre and the Metropolitan Region, 2018 (n=460)

Level	Case Rate/1 thousand inhabitants <18 years	Number of cases	SVI	HDI	Population aged <18 years
Municipality (n=34)					
Highest rates	4.05	32	0.163	0.721	7,910
Igrejinha	2.52	769	0.249	0.805	305,103
Porto Alegre	2.51	12	0.207	0.688	4,790
Rolante	2.38	3	0.226	0.679	1,259
Araricá	2.10	52	0.304	0.730	24,738
Guaíba					
Lowest rates	0.48	3	0.140	0.743	6,226
Dois Irmãos	0.41	6	0.142	0.745	14,579
Campo Bom	0.40	24	0.200	0.747	59,286
Novo Hamburgo	0.29	24	0.256	0.750	82,985
Canoas	0.13	9	0.303	0.736	67,589
Gravataí					
Human Development Unit (UDH) (n=426)					
Highest rates	20.83	4	0.407	0.593	192
Restinga: Vale do Salso (Porto Alegre)	15.87	3	0.381	0.618	189
Navegantes: DC Navegantes/Casa de Passagem (Porto Alegre)	11.83	2	0.111	0.853	169
Vila Jardim: Beco Um Salvador Pinheiro/Souza Lobo (Porto Alegre)	10.99	2	0.236	0.736	182
Anchieta (district) (Porto Alegre)	10.64	6	0.317	0.627	564
Vila Nova: Condomínio Cristal II (Porto Alegre)					
Lowest rates	0.22	1	0.080	0.894	4,626
Marechal Rondon/Nossa Senhora das Graças/Estância Velha (Canoas)	0.20	1	0.320	0.693	5,003
Mathias Velho A (Canoas)	0.17	1	0.273	0.750	5,862
Niterói A (Canoas)	0.17	1	0.440	0.600	5,868
Guajuviras A (Canoas)	0.15	1	0.325	0.723	6,514
Elisa/Santa Fé/Olinda (Gravataí)	4.05	32	0.163	0.721	7,910

## Discussion

This study investigated 1,519 children and adolescents who were victims of sexual violence and were assisted at a specialized health service in 2018, and found that most were female and resided in areas with high SVI values and low HDI values. 

Sexual violence was predominantly intrafamilial. At the UDH level, SVI and HDI showed no correlation with the rate of assisted cases, suggesting a non-monotonic, non-linear relationship between the variables. A positive, albeit weak, correlation was identified between the absolute number of cases assisted and SVI, suggesting that areas with greater social vulnerability may record a higher absolute number of sexual violence cases. The number of cases assisted showed a positive correlation with the population aged <18 years, whereas the case-assistance rate showed a negative correlation.

The results corroborate previous findings indicating that, in most cases, the perpetrator is someone known to the victim ^5^ ^,^ ^17^ ^-^ ^19^ and that females are the most affected by sexual violence ^20^. The possibility of significant underreporting among male victims should be considered, influenced by factors such as fear of stigma related to homosexuality, difficulty in recognizing abuse against boys, and the process of denial of the event ^21^. 

Although the results indicate a higher occurrence of intrafamilial violence in contexts of high social vulnerability, this pattern may reflect greater visibility and reporting of cases due to increased contact between these families and public services. In higher socioeconomic strata, factors such as confidentiality, stigma, and access to private networks may favor underreporting and the invisibility of violence ^22^.

In this study, no statistically significant correlation was identified between the rates of cases assisted and the SVI and HDI indicators. However, an investigation of the spatial distribution of reported cases of sexual violence against male children and adolescents in Brazil, in which municipalities were the unit of analysis, identified a concentration of municipalities with high violence rates and low human development indices, especially in Amazonas, Roraima, and Pará ^23^.

The positive correlation identified between the population aged <18 years and the absolute number of attendances for sexual violence was expected, given the larger contingent of individuals in this age group. The negative correlation between this population and the case rate suggested that, proportionally, more populous areas present a lower relative occurrence of attendances. This pattern may indicate barriers to access to health services, as observed in large urban contexts with extensive territory and high population density ^24^.

In this study, the UDH with the highest rate of sexual violence cases per 1 thousand inhabitants aged <18 years was a region with high social vulnerability and low HDI, illustrating this context of social determination. In line with these findings, in three Brazilian capitals, including Porto Alegre, and among individuals aged 18 to 24 years, an inverse relationship was found between the prevalence of sexual violence and family per capita income and educational attainment, especially among women ^25^.

Among the possible limitations of this study, inherent to research using secondary data, is the quality of the records, particularly regarding address information and the victim's race/skin color, which could not be addressed in the analyses due to the high proportion of missing data in the medical records. Another limitation is that population data, SVI, and HDI were derived from the 2010 IBGE census, potentially underestimating the level of vulnerability of the individuals studied ^26^. Investment in improving service recording programs and in new ways of processing these data, such as geoprocessing (i.e., the map shown in Figure 1), may facilitate the work of health professionals and managers when considering territorialization. 

The findings of this study raised important, though not conclusive, reflections on the meaning of care rates for sexual violence among children and adolescents. The high rate of cases in certain UDH may indicate the severity of the local situation and the effectiveness of the protection network and access to specialized services. The fact that 296 UDH had no record of care during the analyzed period should not be interpreted as good news. The absence of cases may reflect difficulties in identifying them or barriers to access to the health service studied.

Primary Health Care (PHC), due to its territorialization and its bond with the enrolled population, is strategic for the contextual interpretation of data, early detection of violence, and referral to specialized services. Nevertheless, only 4.6% of notifications of sexual violence against children and adolescents over five years occurred in the PHC in the state ^27^. For PHC to fulfill this role, strengthening through professional training and overcoming underfunding is essential ^28^.

To the extent possible to identify, this was the first study to analyze the relationship between sexual violence against children and adolescents and SVI and HDI using data from a specialized and referral health service in Rio Grande do Sul. The substantial number of cases included (n=1,519) highlights the service's relevance, as 2,234 case notifications were recorded in 2018 in VIVA ^29^. The use of the SVI is particularly relevant. It has recently gained prominence as one of the criteria adopted to allocate new federal co-financing for PHC, with the aim of a more equitable distribution of resources ^30^.

Finally, the individual-level analysis indicated that most victims resided in more vulnerable locations with lower human development. At the aggregate level, the rate of cases assisted was not correlated with SVI or HDI. With these findings, this study aims to contribute to addressing sexual violence and to improving the SGDCA, taking into account territories and the social determination of health.

## Data Availability

Data were collected directly from the electronic medical records of a referral service for the comprehensive care of children and adolescents who are victims of sexual violence. Although the database is coded to protect individual identities, it includes variables such as sex, age, address, and date of care. Considering these characteristics, given that the sample consists of minors and the sensitive nature of the topic, and to protect individuals and comply with ethical commitments to ensure the confidentiality of personal data, the Research Ethics Committee agreed that the data would not be made available in an open-access repository.

## References

[1] World Health Organization, Pan American Health Organization (2012). Understanding and addressing violence against women: intimate partner violence.

[2] Borges JL, Dell'Aglio DD (2008). Abuso sexual infantil: indicadores de risco e consequências no desenvolvimento de crianças. Interam J Psychol.

[3] Brasil. Ministério da Saúde. Secretaria de Atenção à Saúde. Departamento de Ações Programáticas Estratégicas (2010). Linha de cuidado para a atenção integral à saúde de crianças, adolescentes e suas famílias em situação de violências: orientação para gestores e profissionais de saúde.

[4] World Health Organization (2003). Guidelines for medico-legal care for victims of sexual violence.

[5] Rates SMM, Melo EM, Mascarenhas MDM, Malta DC (2015). Violence against children: an analysis of mandatory reporting of violence, Brazil 2011. Cienc Saude Colet.

[6] Fórum Brasileiro de Segurança Pública (2024). Anuário Brasileiro de Segurança Pública 2024.

[7] Gaspar RS, Pereira MUL (2018). Evolução da notificação de violência sexual no Brasil de 2009 a 2013. Cad Saude Publica.

[8] Ferreira H, Coelho DSC, Cerqueira DRDC, Alves P, Semente M (2023). Elucidando a prevalência de estupro no Brasil a partir de diferentes bases de dados.

[9] Fórum Brasileiro de Segurança Pública (2023). Anuário Brasileiro de Segurança Pública 2023.

[10] Costa MCO, Carvalho RC, Santa Bárbara JFR, Santos CAST, Gomes WA, Sousa HL (2007). O perfil da violência contra crianças e adolescentes, segundo registros de Conselhos Tutelares: vítimas, agressores e manifestações de violência. Cienc Saude Colet.

[11] Brasil (2017). Lei nº 13.431, de 4 de abril de 2017. Estabelece o sistema de garantia de direitos da criança e do adolescente vítima ou testemunha de violência.

[12] Brunello MEF, Chiaravalloti Neto F, Arcêncio RA, Andrade RLP, Magnabosco GT, Villa TCS (2011). Áreas de vulnerabilidade para co-infecção HIV/aids-TB em Ribeirão Preto, SP. Rev Saude Publica.

[13] Calistro MO, Teixeira Y, Lacerda IRAS, Sousa SM, Neto JA, Duavy SMP (2021). Territorialização com uso de georreferenciamento e estratificação de vulnerabilidade social familiar na Atenção Básica. Cienc Saude Colet.

[14] STROBE Initiative Strengthening the reporting of observational studies in epidemiology (STROBE).

[15] Eloy CB (2012). A credibilidade do testemunho da criança vítima de abuso sexual no contexto judiciário. Psicol Cienc Prof.

[16] Marguti BO, Rocha BN, Pinto CVS, Costa MA, Curi RLC (2018). Métodos e conceitos para o cálculo do Índice de Vulnerabilidade Social com base nas PNADs e desagregações: relatório institucional.

[17] Cerqueira D, Coelho D (2014). Estupro no Brasil: uma radiografia segundo os dados da saúde (versão preliminar).

[18] Platt VB, Back IDC, Hauschild DB, Guedert JM (2018). Violence sexual contra crianças: autores, vítimas e consequências. Ciênc. Saúde Colet.

[19] Trindade LC, Linhares SMGM, Vanrell J, Godoy D, Martins CA, Barbas SMAN (2014). Sexual violence against children and vulnerability. Rev Assoc Med Bras.

[20] Reddock R, Reid SD, Nickenig T (2022). Child sexual abuse and the complexities of gender, power, and sexuality. J Interpers Violence.

[21] Watkins B, Bentovim A (1992). The sexual abuse of male children and adolescents: a review of current research. J Child Psychol Psychiatry.

[22] Pires TO, Silva ATC (2018). Violência intrafamiliar: invisibilidade entre as classes sociais mais altas. Rev Psicol Teor Prat.

[23] Lima BCL, Silva FLD, Souza ER, Ribeiro AP, Lima ACS (2024). Análise temporal e espacial das notificações de violência sexual contra crianças e adolescentes do sexo masculino no Brasil, 2013 a 2022: estudo ecológico. Epidemiol Serv Saude.

[24] Oliveira TS, Pereira AMM (2024). Expressions of inequalities in access to health services in Latin America: a scoping review. Cienc Saude Colet.

[25] Moraes CL, Cabral CS, Heilborn ML (2006). Magnitude e caracterização de situações de coerção sexual vivenciadas por jovens de três grandes capitais brasileiras: Porto Alegre, Rio de Janeiro e Salvador. Cad Saude Publica.

[26] Brasil. Ministério da Saúde. Secretaria de Vigilância em Saúde. Departamento de Vigilância de Doenças e Agravos Não Transmissíveis e Promoção da Saúde (2019). Saúde Brasil 2018: uma análise de situação de saúde e das doenças e agravos crônicos: desafios e perspectivas.

[27] Lourenço SS, Souza MT, Dias R, Costa MS, Pereira TS (2023). Notificações de violência sexual contra crianças e adolescentes no Rio Grande do Sul: estudo descritivo, 2014-2018. Epidemiol Serv Saude.

[28] Massuda A, Fernandez M, Kemper ES, Paschoalotto MAC, Tapia R (2024). Análise de políticas de investimento na Atenção Primária à Saúde: neoinstitucionalismo histórico aplicado ao sistema de saúde brasileiro. Rev Adm Publica.

[29] Brasil. Ministério da Saúde Sistema de Informação de Agravos de Notificação – SINANNet.

[30] Brasil. Ministério da Saúde (2024). Nota Técnica nº 122/2024-CGFAP/SAPS/MS.

