# Supplementary material for: Social vulnerability of children and adolescents who are victims of sexual violence assisted in a specialized service: descriptive study, Porto Alegre and metropolitan region, 2018
Source: Epidemiol Serv Saude. 2026 Oct 30;35:e20240780. doi: 10.1590/S2237-96222026v35e20240780.en (PMC13686305; doi:10.1590/S2237-96222026v35e20240780.en)
Supplement: Supplementary file 1 [file 2237-9622-ress-35-e20240780-sup-en.pdf]

Supplementary Material

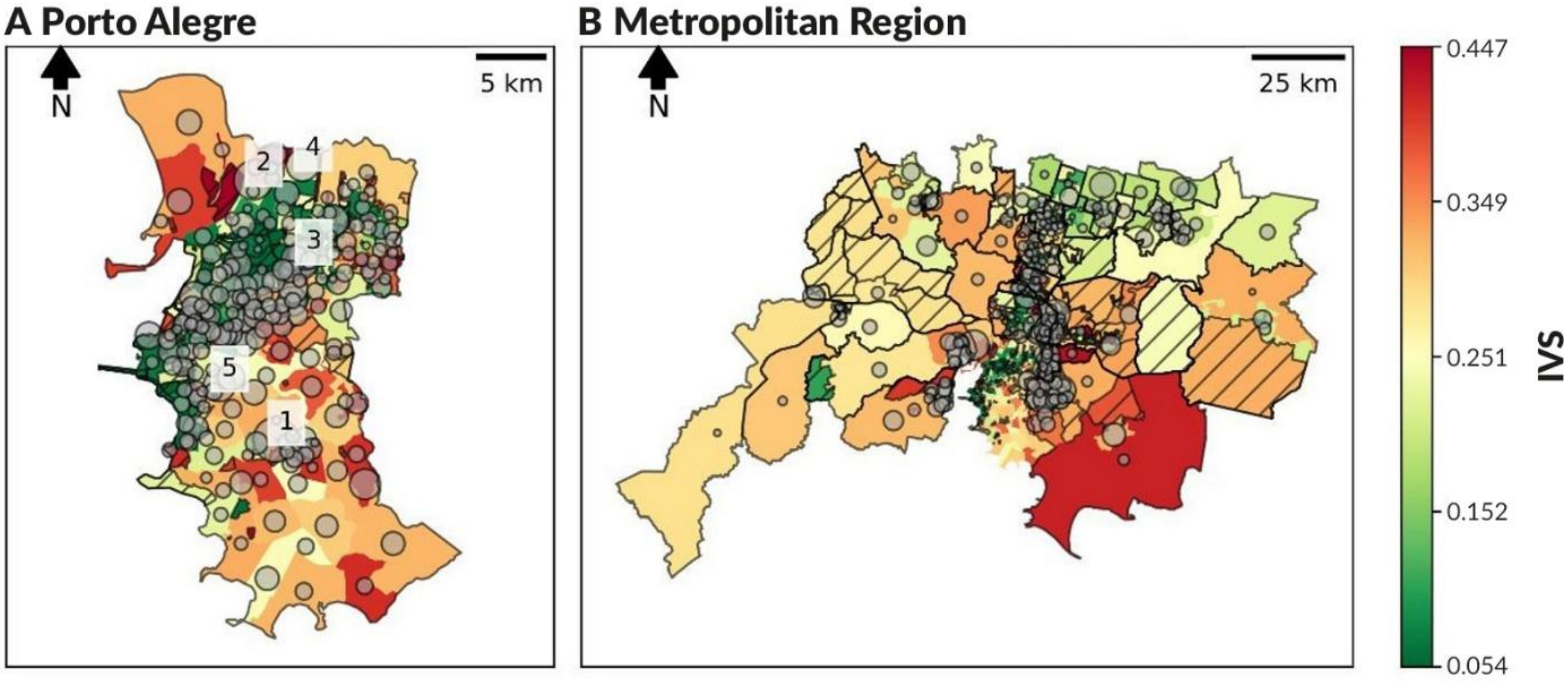

Figure 1. Case rate per 1 thousand inhabitants aged <18 years, by Human Development Unit (UDH) and their respective Social Vulnerability Index (SVI) values in Porto Alegre (A) and Metropolitan Region (B). Porto Alegre and the Metropolitan Region, 2018

Note: Gray circles represent the rate of cases assisted per 1,000 inhabitants aged <18 years in the Human Development Units (UDH), with size varying according to the magnitude of the rates. The color gradient of each UDH corresponds to its SVI value, as indicated in the color legend on the right side of the figure. UDH with no cases assisted is represented with hatched shading. Black outlines indicate municipal boundaries. The numbers on the map indicate the region where the five human development units with the highest case rates are located, all of them belonging to Porto Alegre, listed below: 1- Restinga: Vale do Salso; 2- Navegantes: DC Navegantes/Casa de Passagem; 3- Vila Jardim: Beco Um Salvador Pinheiro/Souza Lobo; 4- Anchieta (district); 5- Vila Nova: Condomínio Cristal II (Porto Alegre).
