# Supplementary material for: Social vulnerability of children and adolescents who are victims of sexual violence assisted in a specialized service: descriptive study, Porto Alegre and metropolitan region, 2018
Source: Epidemiol Serv Saude. 2026 Oct 30;35:e20240780. doi: 10.1590/S2237-96222026v35e20240780.en (PMC13686305; doi:10.1590/S2237-96222026v35e20240780.en)
Supplement: Supplementary file 2 [file 2237-9622-ress-35-e20240780-sup-pt.pdf]

Material suplementar

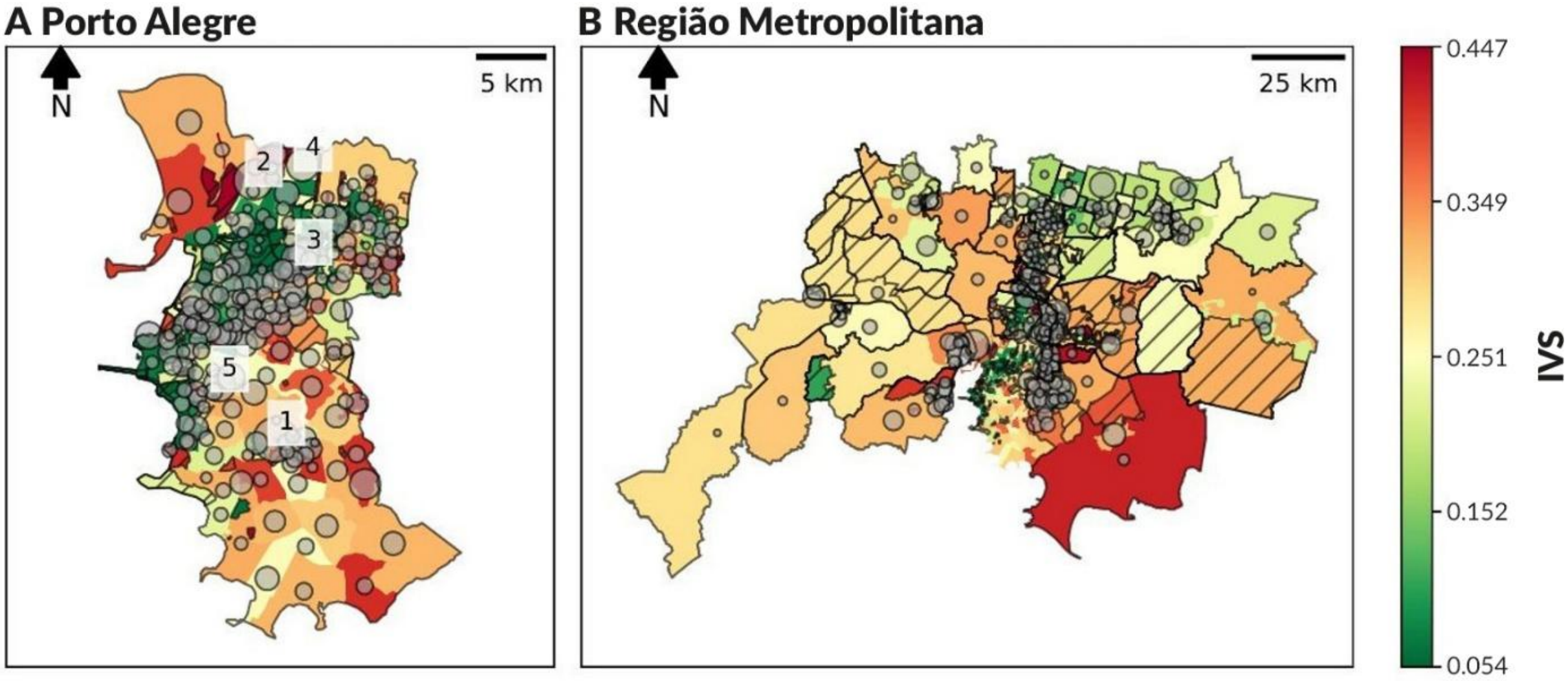

Figura suplementar 1. Taxa de casos/1 mil habitantes <18 anos, segundo unidade de desenvolvimento humano e seus respectivos valores de índice de desenvolvimento humano (IDH) em Porto Alegre (A) e na sua região metropolitana (B). Porto Alegre e região metropolitana, 2018

Nota: Os círculos cinzas representam a taxa de casos atendidos por 1 mil habitantes <18 anos das unidades de desenvolvimento humano e variam de tamanho conforme a magnitude das taxas. A variação de cores de cada unidade de desenvolvimento humano representa seus respectivos valores de índice de desenvolvimento humano (IDH), conforme legenda de gradiente de cor presente ao lado direito da figura. As unidades de desenvolvimento humano que não tiveram casos atendidos estão representadas com preenchimento tracejado. As delimitações geográficas com contorno preto são referentes aos municípios. Os números no mapa indicam a região onde se encontram as cinco unidades de desenvolvimento humano com as maiores taxas de casos, todas elas pertencentes a Porto Alegre: 1- Restinga: Vale do Salso; 2- Navegantes: DC Navegantes/Casa de Passagem; 3- Vila Jardim: Beco Um Salvador Pinheiro/Souza Lobo; 4- Anchieta (bairro); 5- Vila Nova: Condomínio Cristal II (Porto Alegre).
