# Supplementary material for: Social vulnerability of children and adolescents who are victims of sexual violence assisted in a specialized service: descriptive study, Porto Alegre and metropolitan region, 2018
Source: Epidemiol Serv Saude. 2026 Oct 30;35:e20240780. doi: 10.1590/S2237-96222026v35e20240780.en (PMC13686305; doi:10.1590/S2237-96222026v35e20240780.en)
Supplement: Translation

# Translation: Vulnerabilidade social de crianças e de adolescentes vítimas de violência sexual atendidas em serviço especializado: estudo descritivo, Porto Alegre e região metropolitana, 2018

**DOI:** 10.1590/S2237-96222026v35e20240780.pt

**Authors:** Beatriz Raffi Lerm, Vanessa Pletsch, Maria De Fátima Fernandes Géa, Iná da Silva dos Santos, Andrea Pereira Regner, Camila Giugliani

**Affiliations:** 1 Universidade Federal do Rio Grande do Sul, Porto Alegre, RS, Brasil; 2 Hospital Materno Infantil Presidente Vargas, Porto Alegre, RS, Brasil; 3 Universidade Federal de Pelotas, Pelotas, RS, Brasil

**Keywords:** Abuso Sexual na Infância, Delitos Sexuais, Mapeamento Geográfico, Adolescente, Estudos Transversais.

## Abstract

**Objetivo:**

Descrever o perfil e o contexto social de crianças e adolescentes vítimas de violência sexual, bem como os seus territórios de residência, atendidas em 2018 por serviço de referência em Porto Alegre e na sua região metropolitana.

**Métodos:**

Estudo descritivo, com análise em nível individual e agregado, com a utilização de geoprocessamento. Foram avaliados o índice de vulnerabilidade social e o índice de desenvolvimento humano das unidades de desenvolvimento humano de residência das vítimas. Em nível individual, os índices foram comparados com a média das unidades de desenvolvimento humano de Porto Alegre e da sua região metropolitana. No nível agregado, calculou-se a taxa de casos atendidos por 1 mil habitantes <18 anos e utilizou-se a correlação de Spearman e a representação por mapa coroplético.

**Resultados:**

Foram incluídas 1.519 crianças e adolescentes, majoritariamente meninas (77,7%) e vítimas de violência intrafamiliar (53,9%). Em relação à média das unidades de desenvolvimento humano, 63,0% residiam em áreas com índice de vulnerabilidade social mais elevado e 68,5% em áreas com índice de desenvolvimento humano mais baixo. No nível agregado, a taxa de casos não se correlacionou com os índices analisados. Observou-se correlação fraca entre o índice de desenvolvimento humano e o número de casos (r=0,095; p-valor<0,001).

**Conclusão:**

Em nível individual, a maioria das vítimas residia em locais mais vulneráveis e de menor desenvolvimento humano. Em nível agregado, os resultados não indicaram um padrão. Os achados visam contribuir para o enfrentamento da violência sexual, levando-se em consideração os territórios e a determinação social de saúde.

**Aspectos éticos**

Esta pesquisa respeitou os princípios éticos, obtendo os seguintes dados de aprovação: Comitê de ética em pesquisa: Hospital Materno Infantil Presidente Vargas. Número do parecer: 27707119.1.0000.5329. Data de aprovação: 12/2/2020. Certificado de apresentação de apreciação ética: 3.835.179. Termo de consentimento livre e esclarecido: Dispensado.

## Introdução 

A violência sexual, além de configurar uma violação dos direitos humanos, causa sequelas psicológicas, emocionais e físicas às vítimas, em curto e em longo prazo ^1^, sobretudo quando ocorre nas fases iniciais da vida ^2^. No contexto infantojuvenil, o termo violência sexual corresponde a todo ato ou jogo sexual com a intenção de estimular sexualmente a criança ou o adolescente, com o objetivo de utilizá-lo para obtenção de satisfação sexual, praticado por autores que se encontram em estágio de desenvolvimento psicossexual mais avançado do que a criança ou o adolescente ^3^.

Segundo o modelo ecológico proposto pela Organização Mundial da Saúde, as causas da violência sexual envolvem diversos fatores, que incluem: os de ordem comunitária, como a pobreza e a tolerância da comunidade para eventos de violência sexual; e os de ordem social, como o machismo, a iniquidade de gênero e as normas sociais que naturalizam a violência sexual ^1^ ^,^ ^4^. 

A violência sexual contra menores ocorre, na maioria das vezes, dentro do núcleo familiar, o que contribui para a resistência em denunciar os eventos ^3^. A investigação do tema é especialmente complexa devido aos aspectos éticos, às dificuldades metodológicas e aos tabus associados ^1^, bem como pela subnotificação dos casos ^5^. 

Conforme os dados das ocorrências criminais registradas em 2023, o Rio Grande do Sul ocupa a décima posição entre as 26 unidades federativas brasileiras no que se refere à taxa de estupro de <18 anos, com uma estimativa de 174 casos para cada 100 mil habitantes ^6^. Desde 2011, a notificação de violências pelos serviços de saúde passou a ser obrigatória pelo Sistema de Vigilância de Violências e Acidentes (VIVA), porém, ainda é frequente o não cumprimento dessa medida por parte dos profissionais ^7^. Estima-se que somente 4,2% dos casos de estupro no Brasil são registrados nesse sistema ^8^. 

Para uma aproximação da realidade do dimensionamento da violência sexual infantojuvenil, torna-se necessária a utilização de diferentes fontes de dados, como os de boletins de ocorrência ^9^, os de serviços especializados ^10^ e os de bancos de dados oficiais ^7^.

Apesar de ainda representar grave problema de saúde pública no Brasil, o tema obteve avanços na última década, como a Lei nº 13.431/2017, que institui o Sistema de Garantia de Direitos da Criança e do Adolescente vítima ou testemunha de violência, que integra saúde, assistência, segurança pública e justiça ^11^. No âmbito dessa legislação, também é prevista a criação de serviços especializados, com vistas a evitar a revitimização.

A maioria dos estudos brasileiros na área da saúde sobre violência sexual de crianças e de adolescentes baseia-se nos dados do Sinan e possui como enfoque a caracterização do perfil das vítimas, dos agressores e das circunstâncias da violência ^8^. Estudos que investigam os contextos sociais de crianças e de adolescentes vítimas dessa violência ainda são escassos. O geoprocessamento pode contribuir para explorar a relação da violência sexual e da vulnerabilidade social ^12^, o que pode contribuir para o processo de tomada de decisões em saúde pública ^13^.

Este estudo tem como objetivo descrever o perfil e o contexto social de crianças e de adolescentes vítimas de violência sexual, bem como os seus territórios de residência, atendidas em 2018 por serviço de referência em Porto Alegre e na sua região metropolitana.

## Métodos 

Desenho do estudo

Estudo descritivo de dados de registro de serviço de saúde, com análises em nível individual e agregado, com utilização de geoprocessamento de dados. Os dados foram apresentados conforme as diretrizes para estudos observacionais ^14^.

Contexto

Foram utilizados dados de prontuários de serviço destinado ao atendimento biopsicossocial de crianças e de adolescentes vítimas de violência sexual. Tal serviço, sediado em Porto Alegre, Rio Grande do Sul, foi um dos pioneiros na realização do atendimento integral das vítimas - realiza o boletim de ocorrência, os exames periciais, o atendimento biopsicossocial e outros encaminhamentos para a transição de cuidado na rede de proteção -, com vistas a evitar a revitimização por repetidas oitivas em diferentes instituições ^15^.**Participantes**

Os dados de prontuários das crianças e dos adolescentes atendidos no serviço foram extraídos com a consideração apenas dos atendimentos realizados em 2018. Excluíram-se os casos em que a vítima não residia em Porto Alegre ou na região metropolitana da capital.

Coleta e fonte de dados

As variáveis de sexo, de data de nascimento, de endereço de moradia e de vínculo com o abusador foram coletadas em 2020, a partir de prontuários médicos eletrônicos. Os dados foram submetidos à dupla digitação em uma planilha do programa Microsoft Office Excel de 2016, versão 16.0, e codificados, a fim de preservar o sigilo de identidade dos indivíduos envolvidos. **Variáveis**

A idade, expressa como um número fracionário de anos, foi calculada através da diferença entre a data de atendimento e a data de nascimento. A partir desta, foram geradas cinco categorias de faixa etária em anos (<1; 1-5; 6-9; 10-14; 15-18). A variável sexo foi categorizada em feminino e masculino. Em relação ao local de moradia, foi gerada uma variável dicotômica distinguindo as crianças moradoras de Porto Alegre das crianças que habitavam a sua região metropolitana. O vínculo da vítima com o perpetrador da violência foi categorizado em: intrafamiliar (pai, mãe, padrasto, madrasta, irmão(ã), avô(avó), companheiro da avó, tio(a), cunhado(a), primo(a), bisavô(ó), sobrinho(a) e enteado da mãe); extrafamiliar (namorado(a), amigo(a), conhecido ou colega na instituição social em que vivia a vítima - instituições de acolhimento, abrigos, casas lares e orfanatos); intra e extrafamiliar (quando a mesma criança apresentou ao menos um envolvido de cada grupo citado anteriormente); e indefinido (quando nenhum perpetrador foi indicado). Foram geradas duas variáveis dicotômicas: uma referente ao número de perpetradores envolvidos, com distinção das vítimas que relataram um perpetrador daquelas que relataram dois ou mais perpetradores envolvidos no ocorrido; e outra indicando se a violência foi intrafamiliar. 

O índice de vulnerabilidade social (IVS) é calculado a partir da média aritmética dos índices das dimensões de infraestrutura urbana, capital humano e renda e trabalho. Esse índice varia de 0 a 1 e é importante destacar que possui polaridade negativa: quanto mais próximo de 1, maior é a vulnerabilidade social, ou seja, piores são as condições observadas. Já o índice de desenvolvimento humano (IDH) é obtido por meio da média geométrica dos índices de renda, educação e longevidade e também varia entre 0 e 1. Diferentemente do IVS, o IDH apresenta polaridade positiva, o que indica que valores mais próximos de 1 refletem melhores condições de desenvolvimento humano ^16^.

Embora o IVS e o IDH apresentem pontos de corte e categorias previamente definidas por seus criadores, neste estudo, optou-se por classificá-los com base em quintis gerados a partir dos valores de todas as unidades de desenvolvimento humano de Porto Alegre e da sua região metropolitana. Optou-se pela classificação em quintis por indicar melhor as disparidades nesses locais, tendo em vista que os índices são calculados em nível nacional. 

O IVS apresentou os seguintes pontos de corte: 1º quintil (≤0,176), 2º quintil (>0,176 a ≤0,230), 3º quintil (>0,230 a ≤0,286), 4º quintil (>0,286 a ≤0,344) e 5º quintil (>0,344). Os quintis de IDH foram definidos como 1º quintil (≤0,644), 2º quintil (>0,644 a ≤0,704), 3º quintil (>0,704 a ≤0,739), 4º quintil (>0,739 a ≤0,828) e 5º quintil (>0,828). 

Foram construídas duas variáveis dicotômicas para indicar se a vítima residia em uma unidade de desenvolvimento humano com estimativas do IVS e do IDH superiores à média dos índices de todas as unidades de desenvolvimento humano de Porto Alegre e da sua região metropolitana, sendo o IVS de 0,234 e o IDH de 0,746.

Geoprocessamento

Esta etapa da análise foi realizada através da biblioteca de processamento geoespacial Geopandas, em linguagem Python (disponível em: https://geopandas.org/). Primeiramente, as coordenadas exatas dos endereços das vítimas foram obtidas com o auxílio do serviço de geocodificação do Google Maps. A partir delas, localizaram-se as respectivas áreas de unidade de desenvolvimento humano, que são delimitações geográficas das capitais e das suas áreas metropolitanas, elaboradas a partir da homogeneidade socioeconômica e definidas com base nos setores censitários do Instituto Brasileiro de Geografia e Estatística de 2010.

A delimitação geoespacial dessas áreas e dos municípios, assim como as respectivas estimativas do IVS, do IDH e da população <18 anos, são disponibilizadas pelo Instituto de Pesquisa Econômica Aplicada (IPEA) por meio da plataforma digital acessível em: http://ivs.ipea.gov.br. Utilizou-se a unidade de desenvolvimento humano por ser o nível mais desagregado disponível no IPEA, o que permitiu maior aproximação da realidade socioeconômica e captou desigualdades intraurbanas relevantes. 

Posteriormente, foi gerado um mapa coroplético em nível de unidade de desenvolvimento humano, com gradiente de cor que sinalizava a magnitude do IVS. A taxa de casos atendidos por 1 mil habitantes <18 anos de cada unidade foi plotada, e o tamanho dos pontos variou de acordo com a magnitude das taxas. As unidades de desenvolvimento humano que não tiveram nenhum caso atendido foram representadas no mapa (Figura 1) com preenchimento tracejado. Elaborou-se um mapa com os valores do IDH, disponibilizado no material suplementar (Figura suplementar 1).

Análise de dados

Em nível individual, os casos de violência intrafamiliar (exclusivos ou em combinação com violência extrafamiliar) foram descritos separadamente. A idade das vítimas foi descrita por meio de média e de desvio-padrão (DP). Para a comparação da média de idade entre os sexos, utilizou-se o teste t de Student. A relação entre as variáveis dicotômicas e categóricas foi verificada por meio do teste exato de Fisher.

Nos níveis agregados de unidade de desenvolvimento humano e de municípios, a taxa de crianças e de adolescentes atendidos em 2018 foi calculada a partir da divisão do número de casos atendidos em cada unidade de desenvolvimento humano pela população local <18 anos, com a multiplicação do resultado por 1 mil habitantes. 

Em nível de unidade de desenvolvimento humano, foram correlacionadas as variáveis de taxas de casos por 1 mil habitantes, o número de casos, a população <18 anos, o IVS e o IDH, com a utilização da correlação de postos de Spearman, tendo em vista a distribuição assimétrica das variáveis. O nível de significância foi estabelecido como p-valor<0,050. O programa estatístico utilizado foi o Stata, versão 16.1.

**Figura 1:** Taxa de casos/1 mil habitantes <18 anos, segundo unidade de desenvolvimento humano e seus respectivos valores de índice de vulnerabilidade social (IVS) em Porto Alegre (A) e na sua região metropolitana (B). Porto Alegre e região metropolitana, 2018

Nota: Os círculos cinzas representam a taxa de casos atendidos por 1 mil habitantes <18 anos das unidades de desenvolvimento humano, variando de tamanho conforme a magnitude das taxas. A variação de cores de cada unidade de desenvolvimento humano representa seus respectivos valores de índice de vulnerabilidade social (IVS), conforme legenda de gradiente de cor presente ao lado direito da figura. As unidades de desenvolvimento humano que não tiveram casos atendidos estão representadas com preenchimento tracejado. As delimitações geográficas com contorno preto são referentes aos municípios. Os números no mapa indicam a região onde se encontram as cinco unidades de desenvolvimento humano com as maiores taxas de casos, todas elas pertencentes a Porto Alegre, elencadas a seguir: 1- Restinga: Vale do Salso; 2- Navegantes: DC Navegantes/Casa de Passagem; 3- Vila Jardim: Beco Um Salvador Pinheiro/Souza Lobo; 4- Anchieta (bairro); 5- Vila Nova: Condomínio Cristal II (Porto Alegre).

## Resultados 

Em 2018, foram atendidas 1.913 crianças e adolescentes no serviço de saúde estudado. Destes, 1.519 eram moradores de Porto Alegre ou da sua região metropolitana e constituíram a amostra final deste estudo. Dentre as não incluídas, 379 eram provenientes de outros municípios, dez não possuíam informação de endereço nos seus registros e para cinco indivíduos não foi possível encontrar o endereço na análise de geoprocessamento. A média de idade da população estudada foi de dez anos (DP 4,26). A das meninas foi de 10,3 anos (DP 4,29), maior do que a dos meninos, de 8,8 anos (DP 3,93; p-valor<0,001). A faixa etária predominante foi 10 a 14 anos (40,9%).

Em 42 casos (2,8%), houve dois ou mais envolvidos no ato da violência sexual. A violência do tipo intrafamiliar foi a mais frequente na população em estudo, 53,3%. A proporção de meninas que sofreram violência sexual intrafamiliar foi maior, 56,3%, quando comparada com a de meninos, 43,1% (p-valor<0,001). 

A maioria das vítimas (63,0%) residiam em regiões com valores de índice de vulnerabilidade social (IVS) superiores à estimativa estadual, e 31,5% residiam em locais com índice de desenvolvimento humano (IDH) acima do valor do restante das unidades de desenvolvimento humano. 

Foi observada uma maior proporção de vítimas nos dois quintis de maior IVS, 47,8%, sem diferenças estatisticamente significativas entre os sexos. As maiores proporções em relação ao IDH se deram no segundo e no terceiro quintis, 47,3%. Identificou-se diferença estatisticamente significativa (p-valor 0,002) entre os sexos: a maior proporção de meninas foi encontrada no segundo quintil, 25,9%, enquanto entre os meninos predominou o quintil de maior desenvolvimento humano, 22,4% (Tabela 1).

**Tabela 1:** Frequência (n) e percentual (%) das características sociodemográficas e do ato da violência sexual contra crianças e adolescentes atendidos no serviço de saúde. Porto Alegre e região metropolitana (RM), 2018 (n=1.519)

Características	Total	Feminino (n=1.180)	Masculino (n= 339)	p-valor^a^
	n (%)	n (%)	n (%)	
Faixa etária (anos)				<0,001
<1	7 (0,5)	5 (0,4)	2 (0,6)	
1-5	342 (22,5)	247 (20,9)	95 (28,0)	
6-9	363 (23,9)	252 (21,4)	111 (32,7)	
10-14	622 (40,9)	511 (43,3)	111 (32,7)	
15-18	185 (12,2)	165 (14,0)	20 (5,9)	
Local de moradia				0,020
Porto Alegre	770 (50,7)	581 (49,2)	189 (55,8)	
Região Metropolitana	749 (49,3)	599 (50,8)	150 (44,2)	
Número de envolvidos na perpetração da violência sexual				0,347
Um	1.477 (97,2)	1.150 (97,5)	327 (96,5)	
Dois ou mais	42 (2,8)	30 (2,5)	12 (3,5)	
Vínculo com perpetrador da violência sexual				<0,001
Intrafamiliar	810 (53,3)	664 (56,3)	146 (43,1)	
Extrafamiliar	362 (23,8)	262 (22,2)	100 (29,5)	
Intra e extrafamiliar	9 (0,6)	7 (0,6)	2 (0,6)	
Indefinido	338 (22,3)	247 (20,9)	91 (26,8)	
Quintis de índice de vulnerabilidade social (IVS)				0,268
1^o^	267 (17,6)	196 (16,6)	71 (20,9)	
2^o^	277 (18,2)	212 (18,0)	65 (19,2)	
3^o^	249 (16,4)	192 (16,3)	57 (16,8)	
4^o^	362 (23,8)	291 (24,6)	71 (20,9)	
5^o^	364 (24,0)	289 (24,5)	75 (22,2)	
Quintis de índice de desenvolvimento humano (IDH)				0,002
1^o^	289 (19,1)	215 (18,2)	74 (21,8)	
2^o^	368 (24,2)	306 (25,9)	62 (18,3)	
3^o^	351 (23,1)	280 (23,7)	71(21,0)	
4^o^	257 (16,9)	201 (17,1)	56 (16,5)	
5^o^	254 (16,7)	178 (15,1)	76 (22,4)	
IVS>média de Porto Alegre e RM	957 (63,0)	757 (64,1)	200 (59,0)	0,085
IDH≤média de Porto Alegre e RM	1.041 (68,5)	833 (70,6)	208 (61,4)	0,001

a Teste exato de Fisher.

Entre as vítimas de violência intrafamiliar, 56,5% residiam na região metropolitana (p-valor<0,001). Metade dos indivíduos pertencia aos dois quintis de maiores valores de IVS (p-valor 0,013); em relação ao IDH, a maior proporção de indivíduos pertencia ao segundo quintil (p-valor<0,001) (Tabela 2). 

Dos 33 municípios da região metropolitana de Porto Alegre, 32 registraram atendimento no serviço de saúde. Apenas um município, Glorinha, não foi representado nas análises, por ausência de casos atendidos no período do estudo. Do total de 722 unidades de Porto Alegre e da sua região metropolitana, 296 (40,9%) não apareceram nesta análise. Destas, 128 (43,2%) pertencem a Porto Alegre. 

A análise de correlação foi estatisticamente significativa (p-valor<0,001) entre a taxa de casos atendidos e o número de casos (r=0,371), a taxa de casos atendidos e a população <18 anos (r=-0,565), o número de casos atendidos e a população <18 anos (r=0,498), o número de casos atendidos e o IVS (r=0,095), conforme matriz de correlação (Tabela 3). Não foi encontrado coeficiente de correlação significativo entre os valores de IVS e a taxa de casos de cada unidade de desenvolvimento humano (r=0,010; p-valor 0,843), nem com os valores de IDH (r=0,072; p-valor 0,135). 

De acordo com este achado, pode-se observar no mapa (Figura 1) que nem sempre os maiores círculos - que representam maiores taxas de casos atendidos - estavam posicionados em unidades de desenvolvimento humano de maior IVS. As cinco unidades de desenvolvimento humano que apresentaram as maiores taxas de casos estão localizadas em Porto Alegre e sinalizadas no mapa pelos números de um a cinco. 

A unidade de Restinga/Vale do Salso apresentou a maior taxa (20,83 casos por 1 mil habitantes) e se caracteriza como uma região de alta vulnerabilidade e baixo desenvolvimento humano (Tabela 4). A unidade de desenvolvimento humano com a menor taxa (0,15 casos por 1 mil habitantes) foi a de Elisa/Santa Fé/Olinda, em Gravataí, que apresentou IVS mais elevado e IDH mais baixo do que as médias das demais unidades de desenvolvimento humano. No nível municipal, a maior taxa foi observada em Igrejinha (4,05 casos por 1 mil habitantes), seguida de Porto Alegre (2,52 casos por 1 mil habitantes). As menores taxas foram registradas em Gravataí (0,13 casos por 1 mil habitantes) e em Canoas (0,29 casos por 1 mil habitantes).

**Tabela 2:** Frequência (n) e percentual (%) do sexo e do contexto social de crianças e de adolescentes vítimas de violência sexual intrafamiliar atendidos no serviço de saúde. Porto Alegre e região metropolitana, 2018 (n=819)

Grupos	n (%)	p-valor^a^
Sexo		<0,001
Feminino	671 (81,9)	
Masculino	148 (18,1)	
Local de moradia		<0,001
Porto Alegre	356 (43,5)	
Região metropolitana	463 (56,5)	
Quantis de índice de vulnerabilidade social		0,013
1º	124 (15,1)	
2º	162 (19,8)	
3º	124 (15,1)	
4º	197 (24,1)	
5º	212 (25,9)	
Quintis de índice de desenvolvimento humano		0,001
1º	163 (19,9)	
2º	214 (26,1)	
3º	185 (22,6)	
4º	149 (18,2)	
5º	108 (13,2)	

a Teste exato de Fisher.

**Tabela 3:** Matriz de correlação entre taxa de casos atendidos/1 mil habitantes <18 anos, número de casos atendidos, número de casos atendidos, população <18 anos, índice de vulnerabilidade social (IVS) e índice de desenvolvimento humano (IDH), no nível de unidade de desenvolvimento humano. Porto Alegre e região metropolitana, 2018 (n=426)

	Taxa de casos	Número de casos	População <18 anos	IVS	IDH
Taxa de casos	1,000				
Número de casos	0,371^a^	1,000			
População <18 anos	-0,565^a^	0,498^a^	1,000		
IVS	0,010	0,095^a^	0,044	1,000	
IDH	0,072	-0,056	-0,093	-0,817^a^	1,000

^a^ p-valor <0,001.

**Tabela 4:** Municípios e unidades de desenvolvimento humano que apresentaram as maiores e as menores taxas de casos atendidos/1 mil habitantes <18 anos e seus respectivos valores de índice de vulnerabilidade social (IVS) e de índice de desenvolvimento humano (IDH). Porto Alegre e região metropolitana, 2018 (n=460)

Nível	Taxa de casos/1 mil habitantes <18 anos	Número de casos	IVS	IDH	População <18 anos
Município (n=34)					
Maiores taxas					
Igrejinha	4,05	32	0,163	0,721	7.910
Porto Alegre	2,52	769	0,249	0,805	305.103
Rolante	2,51	12	0,207	0,688	4.790
Araricá	2,38	3	0,226	0,679	1.259
Guaíba	2,10	52	0,304	0,730	24.738
Menores taxas					
Dois Irmãos	0,48	3	0,140	0,743	6.226
Campo Bom	0,41	6	0,142	0,745	14.579
Novo Hamburgo	0,40	24	0,200	0,747	59.286
Canoas	0,29	24	0,256	0,750	82,985
Gravataí	0,13	9	0,303	0,736	67.589
Unidade de desenvolvimento humano (n=426)					
Maiores taxas					
Restinga: Vale do Salso (Porto Alegre)	20,83	4	0,407	0,593	192
Navegantes: DC Navegantes/Casa de Passagem (Porto Alegre)	15,87	3	0,381	0,618	189
Vila Jardim: Beco Um Salvador Pinheiro/Souza Lobo (Porto Alegre)	11,83	2	0,111	0,853	169
Anchieta (bairro) (Porto Alegre)	10,99	2	0,236	0,736	182
Vila Nova: Condomínio Cristal II (Porto Alegre)	10,64	6	0,317	0,627	564
Menores taxas					
Marechal Rondon/Nossa Senhora das Graças/Estância Velha (Canoas)	0,22	1	0,080	0,894	4.626
Mathias Velho A (Canoas)	0,20	1	0,320	0,693	5.003
Niterói A (Canoas)	0,17	1	0,273	0,750	5.862
Guajuviras A (Canoas)	0,17	1	0,440	0,600	5.868
Elisa/Santa Fé/Olinda (Gravataí)	0,15	1	0,325	0,723	6.514

## Discussão 

Este estudo investigou 1.519 crianças e adolescentes vítimas de violência sexual atendidas em serviço de saúde especializado em 2018 e verificou-se que a maioria era do sexo feminino, residia em locais com elevados índices de vulnerabilidade social (IVS) e com baixos índices de desenvolvimento humano (IDH). 

A violência sexual foi majoritariamente do tipo intrafamiliar. Em nível de unidade de desenvolvimento humano, o IVS e o IDH não apresentaram correlação com a taxa de casos atendidos, o que sugeriu relação não monotônica e não linear entre as variáveis. Identificou-se correlação positiva, ainda que fraca, entre o número absoluto de casos atendidos e o IVS, o que sugeriu que áreas com maior vulnerabilidade social podem registrar mais casos de violência sexual em valores absolutos. O número de casos atendidos apresentou correlação positiva com a população <18 anos, enquanto a taxa de casos atendidos obteve uma correlação negativa.

Os resultados encontrados corroboram achados prévios, de que o perpetrador é, na maioria dos casos, alguém conhecido da vítima ^5^ ^,^ ^17^ ^-^ ^19^ e que o sexo feminino é o mais atingido pela violência sexual ^20^. Cabe considerar a possibilidade de significativo sub-relato entre as vítimas do sexo masculino, influenciado por fatores como o medo do estigma da homossexualidade, a dificuldade em reconhecer abusos contra meninos e o processo de negação do ocorrido ^21^. 

Embora os resultados indiquem maior ocorrência de violência intrafamiliar em contextos de alta vulnerabilidade social, esse padrão pode refletir maior visibilidade e notificação dos casos, devido ao maior contato dessas famílias com serviços públicos. Em camadas socioeconômicas mais altas, fatores como sigilo, estigma e acesso a redes privadas podem favorecer a subnotificação e a invisibilidade da violência ^22^.

Não foi identificada, neste estudo, correlação estatisticamente significativa entre as taxas de casos atendidos e os indicadores de IVS e de IDH. Já em investigação da distribuição espacial dos casos notificados de violência sexual contra crianças e adolescentes do sexo masculino no Brasil, na qual a unidade de análise foram os municípios, identificou-se concentração de municípios com altas taxas de violência e com baixos índices de desenvolvimento humano, especialmente no Amazonas, em Roraima e no Pará ^23^.

A correlação positiva identificada entre a população <18 anos e o número absoluto de atendimentos por violência sexual era esperada, dado o maior contingente de indivíduos nesse grupo etário. A correlação negativa entre essa população e a taxa de casos sugeriu que, proporcionalmente, áreas mais populosas apresentam menor ocorrência relativa de atendimentos. Esse padrão pode indicar barreiras de acesso aos serviços de saúde que podem ser observadas em contextos urbanos de grande extensão e de alta densidade populacional ^24^.

Neste estudo, a unidade de desenvolvimento humano com maior taxa de casos de violência sexual por 1 mil habitantes <18 anos foi uma região de alta vulnerabilidade social e de baixo IDH, o que ilustrou esse contexto de determinação social. Em consonância com tais achados, em três capitais brasileiras, entre elas Porto Alegre, e em indivíduos entre 18 e 24 anos, encontrou-se relação inversa entre a prevalência de violência sexual e a renda per capita da família e a escolaridade, sobretudo entre as mulheres ^25^.

Entre as possíveis limitações deste estudo, inerentes a pesquisas com dados secundários, está a qualidade dos registros, sobretudo no que se diz respeito à informação de endereços e à raça/cor da pele da vítima, o que não foi possível ser abordado nas análises devido à alta proporção de não preenchimento nos prontuários. Outra limitação é que os dados populacionais, o IVS e o IDH foram oriundos do último censo do Instituto Brasileiro de Geografia e Estatística, realizado em 2010, sendo possível que os resultados tenham subestimado o nível de vulnerabilidade dos indivíduos pesquisados ^26^. O investimento na melhoria dos programas de registro dos serviços e em novas formas de processá-los, como a utilização de geoprocessamento (i.e. o mapa da Figura 1), pode facilitar a atuação de trabalhadores e de gestores da saúde ao considerarem a territorialização. 

Os achados deste estudo suscitaram reflexões importantes, mas não conclusivas acerca do significado das taxas de atendimento por violência sexual em crianças e em adolescentes. A elevada taxa de casos em determinadas unidades de desenvolvimento humano pode indicar a gravidade da situação local e a efetividade da rede de proteção e do acesso ao serviço especializado. O fato de 296 unidades de desenvolvimento humano não apresentarem nenhum registro de atendimento durante o período analisado não deve ser interpretado como uma boa notícia. A ausência de casos pode refletir dificuldades na identificação deles ou barreiras no acesso ao serviço de saúde estudado.

A atenção primária à saúde (APS), por sua territorialização e seu vínculo com a população adscrita, é estratégica para a leitura contextual dos dados, a detecção precoce da violência e o encaminhamento a serviços especializados. Mesmo assim, apenas 4,6% das notificações de violência sexual contra crianças e adolescentes em cinco anos ocorreram na APS no estado ^27^. Para que cumpra esse papel, é essencial o fortalecimento da APS através de capacitação profissional e de superação do subfinanciamento ^28^.

Até onde foi possível identificar, este foi o primeiro estudo a analisar a relação entre violência sexual contra crianças e adolescentes e o IVS e o IDH, com dados de serviço de saúde especializado e de referência no Rio Grande do Sul. O expressivo volume de casos incluídos (n=1.519) evidenciou a relevância do serviço, levando-se em consideração que houve 2.234 notificações de casos em 2018 registrados no Sistema de Vigilância de Violências e Acidentes ^29^. A utilização do IVS é particularmente relevante e ganhou recente destaque por compor um dos critérios adotados para definir o repasse do novo cofinanciamento federal da APS, com vistas à distribuição mais equânime dos recursos ^30^.

A análise em nível individual, por fim, indicou que a maioria das vítimas residiam em locais mais vulneráveis e de menor desenvolvimento humano. Em nível agregado, a taxa de casos atendidos não apresentou correlação com o IVS e o IDH. Com esses achados, objetiva-se contribuir para o enfrentamento da violência sexual e o aprimoramento do Sistema de Garantia de Direitos da Criança e do Adolescente, com a consideração dos territórios e da determinação social de saúde.

## Data Availability

Os dados foram coletados diretamente do prontuário eletrônico de serviço de referência para o atendimento integral de crianças e de adolescentes vítimas de violência sexual. Apesar de o banco de dados ser codificado quanto à identidade dos indivíduos, constam variáveis de: sexo, idade, endereço e data de atendimento. Considerando-se essas informações, sendo a amostra composta por menores e a temática sensível, para proteção dos indivíduos e respeito aos compromissos éticos assumidos na garantia de sigilo de dados pessoais, foi pactuado com o Comitê de Ética em Pesquisa que os dados não seriam disponibilizados em repositório aberto.

## Material suplementar

Material suplementar Material suplementar
